# A population-based analysis of the risk of drug interaction between clarithromycin and statins for hospitalisation or death

**DOI:** 10.1186/s12944-015-0134-y

**Published:** 2015-10-24

**Authors:** Bita Mesgarpour, Ghazaleh Gouya, Harald Herkner, Berthold Reichardt, Michael Wolzt

**Affiliations:** Department of Clinical Pharmacology, Allgemeines Krankenhaus Wien, Medical University of Vienna, Währinger Gürtel 18-20, 1090 Vienna, Austria; Digestive Diseases Research Institute (DDRI), Tehran University of Medical Sciences (TUMS), Tehran, Iran; Department of Emergency Medicine, Medical University of Vienna, Vienna, Austria; Sickness Fund Burgenland, Eisenstadt, Austria

**Keywords:** HMG-CoA reductase inhibitors, Drug interactions, Clarithromycin, Electronic health records, Cohort studies

## Abstract

**Background:**

Clarithromycin, known as a potent inhibitor of the cytochrome P450 isoenzyme CYP3A, may increase the plasma concentration of statins metabolized by this pathway; therefore, increase the risk of interaction with statins in reference to pharmacokinetic studies. This study aimed to characterize whether the concomitant use of a statin with clarithromycin is associated with serious outcomes among adult persons.

**Methods:**

Health claims data of adult persons in the Regional Sickness Fund of Burgenland, Austria, who filled a prescription for clarithromycin between July 1, 2009 and June 30, 2012 were reviewed retrospectively. We assumed that the risk of hospitalisation increases acutely with the indication for taking an antibiotic, whereas statin use can be considered a chronic exposure with a low constant effect on hospitalisation. When defining the population as persons taking clarithromycin and the use of statins as the exposure we could achieve a comparable effect in both groups from the acute condition on hospitalisation. Therefore, we defined exposed patients as those who had overlapping treatment with a statin and unexposed controls as those who had filled a prescription for clarithromycin without concomitant statin therapy. Outcome was defined as a composite of hospital admission or death within 30 days after starting clarithromycin. We used generalised linear regression to model an association between outcome and exposure to statins.

**Results:**

Among 28,484 prescriptions of clarithromycin, 2317 persons were co-exposed to statins. Co-administration of CYP3A4 metabolized statins and clarithromycin was associated with a 2.11 fold increased risk of death or hospitalisation (95 % confidence interval [CI]: 1.79–2.48). This effect was explained by age, evidence of cardiovascular disease, diabetes mellitus and utilization of other antibiotics (multivariable adjusted risk ratio: 1.02, 95 % CI: 0.85–1.22). The sensitivity analyses did not change the significance of effect.

**Conclusions:**

The risk for hospitalisation or death in persons receiving clarithromycin increases with age and cardiovascular disease but is not causally associated with statin-clarithromycine co-administration.

**Electronic supplementary material:**

The online version of this article (doi:10.1186/s12944-015-0134-y) contains supplementary material, which is available to authorized users.

## Background

The potential of interaction between drugs that inhibit the CYP3A4 metabolic pathway and 3A4 substrates is well recognised [1–8]. However, the clinical relevance of this interaction is poorly defined and co-prescription of these medications is common in the clinical setting [9–12]. Macrolide antibiotics such as clarithromycin are known as potent inhibitors of CYP3A4, thus introducing the potential for a pharmacological interaction with CYP3A4-metabolised statins (atorvastatin, simvastatin and lovastatin). Concomitant use of macrolides with statins 3A4 substrate was detected from 1.6 to 6.3 % in the electronic medical records databases [9, 11].

Studies showed that co-administration of clarithromycin have significant effect on pharmacokinetic parameters of simvastatin and atorvastatin, with an approximately tenfold or fourfold increase in drug exposure as measured by the area of the drug concentration versus time curve, respectively. It was also associated with approximately seven- to eightfold and greater than fivefold increase in maximum plasma concentration of simvastatin and atorvastatin, respectively [13, 14]. Study on the spontaneous adverse event reports, using the US Food and Drug Administration Adverse Event Reporting System database showed a six fold increase in the adverse event reporting rate and ratio (AERR) of rhabdomyolysis in concomitant use of simvastatin with CYP3A4 inhibitors compared to simvastatin without CYP3A4 inhibitors (56 events in 14,575,000 prescriptions versus 62 events in 103,822,000 prescriptions) [15]. Although, several case reports support this interaction [16–23]. However, limited evidence from controlled studies confirms and measures the potential risk of serious adverse events due to this interaction [9, 24, 25]. Furthermore, none of them has the appropriate power to recognize a specific CYP3A4 inhibitor-statins interaction. In an effort to address the clinical impact of this drug-drug interaction, we did a cohort study to investigate the risk of hospitalization or death associated with co-prescription of a statin with clarithromycin.

## Results

**Fig. 1:**
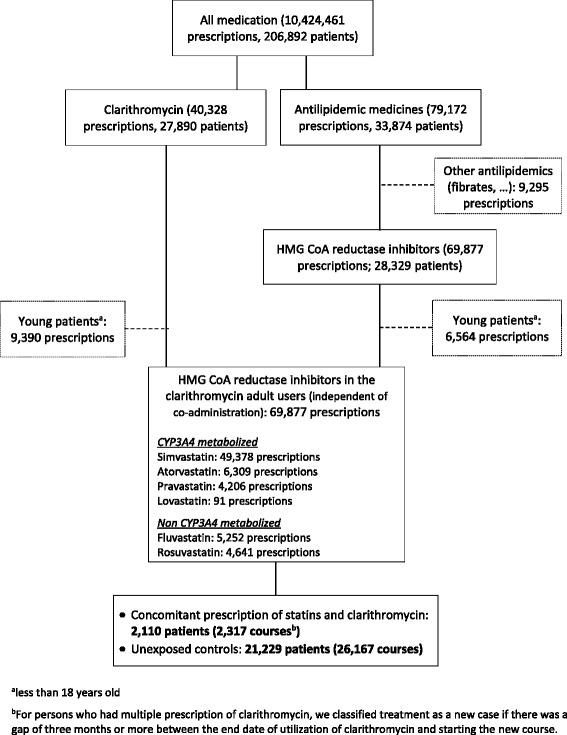
Clarithromycin and antilipidemic agents’ utilization in BGKK from 2009.07.01 to 2012.06.30

In total, 69,877 HMG CoA reductase inhibitors were prescribed in clarithromycin users (independent of co-administration) during the observation period (Fig. 1). A total of 28,484 prescriptions in 23,339 persons were included in the analyses. We determined 2317 co-administrations of statins and clarithromycin as exposed persons comparing to 26,167 prescriptions of clarithromycin only as unexposed control.

Person demographics and prescriptions are presented in Table 1. Among the persons who received co-administrations of statins and clarithromycin, 206 (8.89 %) hospitalizations or deaths were observed during the follow-up period. In the unexposed clarithromycin group, 1128 (4.31 %) persons were hospitalized or died during the observation period.

**Table 1:** Patient demographics and prescriptions

	Exposed patients	Unexposed controls
Age, mean ± SD	66.3 ± 11.1	46.8 ± 17.2
Female	1426 (61.5 %)	16,990 (64.9 %)
Concomitant statins		
*CYP3A4 metabolized*		
Simvastatin	1589	–
Atorvastatin	195	–
Pravastatin	151	–
Lovastatin	2	–
*Non CYP3A4 metabolized*		
Fluvastatin	205	–
Rosuvastatin	175	–
Current statin users	1959	–
History of disease n (%)^a^		
Diabetes	622 (26.8 %)	1236 (4.7 %)
Cardiovascular disease	1906 (82.3 %)	7704 (29.4 %)
Malignancy	52 (2.2 %)	337 (1.3 %)
Autoimmune disorders	27 (1.2 %)	172 (0.7 %)
Antiplatelet drugs	665 (28.7 %)	860 (3.3 %)
P2Y12 inhibitors	194 (8.4 %)	136 (0.5 %)
ASA ± dipyridamole	537 (23.2 %)	757 (2.9 %)
Highly active antiretroviral therapy	0 (0 %)	2 (0 %)
Concomitant medications		
Other antilipidemic agents	116 (5.0 %)	241 (0.9 %)
Other systemic antibiotics	684 (29.5 %)	23,580 (90.1 %)
Drug-drug interaction (synergistic effect)^b^		
Clarithromycin		
Strong interaction	78 (3.4 %)	312 (1.2 %)
Moderate interaction	882 (38.1 %)	2859 (10.9 %)
Weak interaction	581 (25.1 %)	2768 (10.6 %)
Statins		
Strong interaction	22 (0.9 %)	–
Moderate interaction	4 (0.2 %)	–
Weak interaction	21 (0.9 %)	–
Drug-drug interaction (antagonistic effect)^b^		
Clarithromycin	2 (0.1 %)	8 (0.0 %)
Statins	0 (0.0 %)	–
Outcomes		
No. of hospitalization	198 (8.5 %)	1108 (4.2 %)
No. of death	8 (0.3 %)	20 (0.1 %)

*ASA* acetylsalicylic acid, *CYP3A4* cytochrome P450 3A4, *SD* standard deviation

^a^Concomitant disease as suggested by co-medication

^b^See the details in supplementary methods

In the crude unadjusted analysis, current concomitant use of CYP3A4 metabolized statins with clarithromycin was associated with 2.11 (95 % CI: 1.79–2.48) times higher risk for hospitalization or death compared to clarithromycin without current concomitant use of CYP3A4 metabolized statins (Table 2). Current age, treatment with other antibiotics, evidence of diabetes or cardiovascular disease each explained partly the effect of current statins on death or hospitalization in the bivariate analyses. In multivariate combination, however, these four variables fully account for the association between current statin use and the outcome of death or hospitalization (multivariable adjusted RR: 1.02 (0.85–1.22). The effects were virtually unchanged for persons younger or older than 65 years (*p*-value for interaction 0.11). There were also no other significant interactions on the effect of current statin use on the outcome.

**Table 2:** Risk estimate in current concomitant use of CYP3A4 metabolized statins with clarithromycin within 30 days

Risk of death or hospitalisation	RR (95 % CI)	*P* value
Unadjusted risk	2.11 (1.79–2.48)	<0.001
*Adjusted for*		
Current age^a^	1.18 (1.00–1.40)	0.049
Diabetes mellitus	1.60 (1.34–1.91)	<0.001
Cardiovascular disease	1.33 (1.12–1.57)	0.001
Other antibiotics	1.55 (1.28–1.87)	<0.001
*Multivariable adjusted for*		
age, diabetes mellitus, cardiovascular disease and other antibiotic therapy	1.02 (0.85–1.22)	0.85

Estimates come from a generalised linear model using a log link function; confidence intervals are based on robust standard errors

^a^current age as indicator variable of quintiles

We conducted a sensitivity analyses to explore the impact of frequency of clarithromycin use, hospital admission or death within 5 or 10 days and death or hospitalisation separately. Interaction of CYP3A4 metabolized statins and clarithromycin was not significantly different with restriction of exposed persons to those with first time prescription of clarithromycin (RR = 0.96, 95 % CI: 0.79–1.16) or on a one-time basis (RR = 0.92, 95 % CI: 0.74–1.13). Results of the sensitivity analyses for death or hospitalization within 5 or 10 days (RR = 0.78, 95 % CI: 0.55–1.12; RR = 1.38, 95 % CI: 0.95–2.00), only hospitalisation or only death within 30 days (RR = 1.01, 95 % CI: 0.84–1.21; RR = 1.2, 95 % CI: 0.46–3.18) did not differ significantly from our predefined outcome, death or hospitalisation within 30 days.

## Discussion

This population-based analysis of reimbursement data suggest that hospital admission and mortality are similar when clarithromycin is prescribed in the presence or absence of CYP3A4 substrate statins. This finding puts the pharmacokinetic drug interaction into clinical perspective.

Our study suggests a lack of a causal effect for current statin use on death or hospitalisation of clarithromycin users. Increased clinical risk is driven by the effects of age, evidence of diabetes or cardiovascular disease and treatment with other antibiotics.

Our findings are consistent with a population-based cohort study on the UK Health Improvement Network (THIN) database, which detected no difference in the relative hazard of muscle toxicity, renal dysfunction or hepatic dysfunction in patients prescribed a statin CYP3A4 substrate versus a statin non-CYP3A4 substrate in co-medication with another CYP3A4 inhibitor [26]. Our present study extends these findings, since it has focussed on a specific CYP3A4 inhibitor in a large population. Further, we adjusted our data for all possible drug interactions of clarithromycin and statins such as antiretroviral therapy since our data was not limited to general physicians’ prescriptions. The number of prescriptions of some classes of drugs, e.g. antiretroviral treatments, was however too small to draw meaningful conclusions and to rule out relevant clinical effects. We also adjusted for prescription of other systemic antibiotics during the observation period as proxy for the severity of disease.

Likewise, a Canadian population-based, nested case-control study has evaluated drug-drug interaction of donepezil, which is metabolized by CYP3A4, and clarithromycin. The risk of adverse cardiovascular events was not increased by concurrent administration of clarithromycin in elderly donepezil users compared with azithromycin, which does not interact with the cytochrome P-450 system (OR = 0.67; 95 % CI:0.28–1.63; *p* = 0.38) [27]. In contrast, a recent Canadian population-based cohort study detected a higher risk for hospitalization with rhabdomyolysis (RR = 2.17, 95 % CI:1.04–4.53), acute kidney injury (RR = 1.78, 95 % CI, 1.49–2.14) and for all-cause mortality (RR = 1.56, 95 % CI: 1.36–1.80) in older people associated with co-prescription of a CYP3A4 metabolized statin with clarithromycin or erythromycin compared with azithromycin. [25] Another Canadian population-based cohort study found that co-prescription of clarithromycin versus azithromycin with a calcium-channel blocker was associated with a higher risk of hospitalization with acute kidney injury (OR = 1.98, 95 % CI: 1.68–2.34) [28].

The result of our study is at variance with a study on dispensed prescriptions of lipid-lowering drugs using administrative claims data from diverse regions in the United States. The increased risk of hospitalization due to myopathy, renal and hepatic adverse medical events has been identified in lipid-lowering drug users with co-administration of CYP3A4 inhibitors (RR = 6.01, 95 % CI: 2.08–17.38; RR = 2.29, 95 % CI: 1.62–3.23; and RR = 2.55, 95 % CI: 1.76–3.70, respectively) [24]. Although, this study has the limitation of a case-control study design, not controlling of confounding variables like history of other disease, as well as lack of subgroup analysis for detecting the effect of statins-CYP3A4 inhibitors interaction. Moreover, monotherapy with statins are generally well tolerated and present a safe profile. Therefore, the choice of analysis, comparing the co-administration of statins and clarithromycin with monotherapy of statins or monotherapy of clarithromycin might affect the risk of serious adverse events of co-administration.

As we conducted a database analysis of prescription data, medication adherence is not accounted for. In addition, the possibility of a transient interruption of statin intake during clarithromycin therapy by physician advice was not accessible from our database. These factors would have concealed a drug-drug interaction.

This study does not indicate an increased risk for hospitalisations or death when clarithromycin is prescribed with statins in a cohort of unselected persons in primary care. The well-described pharmacokinetic interaction of these medicines does not result in an increased precipitation of serious clinical endpoints.

## Methods

### Population

We established a cohort of persons aged 18 years or older in the Regional Sickness Fund of Burgenland (BGKK) who filled a prescription for reimbursement of clarithromycin between July 1, 2009 and June 30, 2012. The Austrian insurance system provides almost complete coverage of health care for all residents. According to their current employment or providence of residence, the membership to the regional Burgenland Sickness Fund is assigned. We received data from outpatient as well as inpatient medical services covered by the health insurance fund, which were stored in the respective anonymised dataset, including demographic data, information on hospital admissions and discharges with primary diagnoses coded using the International Classification of Diseases (ICD) system and drug prescriptions received. The data linkage with vital status of persons was provided by Statistik Austria. There was no ICD information of the extramural medical care included. The date of dispensing clarithromycin served as the date of cohort entry (entry date). For persons who had multiple prescription of clarithromycin, we classified treatment as a new case if there was a gap of 3 months or more between the end date of utilization of clarithromycin and starting the new course. The initial unit of analysis was a course of clarithromycin treatment (accounting for within-person correlation). For a secondary analysis, we handled single persons as the unit of analysis and used the first course of clarithromycin, ignoring further courses of clarithromycin treatment.

For each person, we determined the total duration of clarithromycin treatment using Defined Daily Dose (DDD) information from WHO; this information was used as a covariate in the analyses.

### Exposed persons

We assumed that the risk of hospitalisation increases acutely with the indication for taking an antibiotic, whereas statin use can be considered a chronic exposure with a low constant effect on hospitalisation. When defining the population as persons taking clarithromycin and the use of statins as the exposure we could achieve a comparable effect in both groups from the acute condition on hospitalisation. Given that we were able to adjust for confounding by indication for statin use we assume that this approach enabled us to appropriately examine the drug-drug interaction of statins and clarithromycin. Given the absence of detailed clinical information in the database, we were not able to adjust for factors related to the indication of clarithromycin use, therefore an approach of comparing clarithromycin exposure in a population of statin users appeared less promising. Therefore, we identified persons who had a statin co-prescribed during clarithromycin treatment. We defined exposed persons as those who were treated with clarithromycin and had overlapping treatment with a statin at least for 1 day (concomitant users). We defined ‘current statin users’ if a person had utilized a statin within 5 days before the entry date. Exposure status was stratified by statins metabolized by the enzyme CYP3A4 (atorvastatin, lovastatin, pravastatin, simvastatin) and those metabolized by the CYP2C9 (fluvastatin, rosuvastatin).

### Unexposed persons

Persons that had no overlapping statin exposure during their clarithromycin treatment were determined as controls.

### Outcome

Outcome was defined as a composite of hospital admission or death within 30 days. In separate sensitivity analyses, we assigned hospital admission as endpoint if it happened within 5, 10 and 30 days after starting clarithromycin (entry date).

### Observation time

We defined the exit date as the date of the occurrence of the outcome or the end of the observation time. *Observation time* = *exit date* - *entry date*.

### Covariates

Prescriptions of drugs used to treat diabetes (ATC code A10), cardiovascular disease (ATC code B01, C01, C02, C03, C04, C07, C08, C09), malignancy (ATC code: L01, L02B, L03), autoimmune disorders (ATC code: L04) and other antilipidemic (ATC code: C10AB, C10AC, C10AD, C10AX and C10B) before the entry date used to identify relevant disease in cases and controls. We identified antiplatelet drugs including P2Y12 inhibitors (ATC code: B01AC04, B01AC22, B01AC24), aspirin and combination of aspirin and dipyridamole (ATC code: B01AC06, B01AC30) as proxies for manifestation of cardiovascular disease. Highly active antiretroviral therapy (HAART) used to treat HIV infection has been detected based on recommended guidelines [29, 30]. We defined the person’s history of the above disease if the relevant medications have been prescribed at least 6 month before the cohort entry.

Clarithromycin is metabolized by the enzyme CYP3A4 (CYP3A4 substrate) and acts as an inhibitor of the metabolizing enzyme CYP3A4. Therefore, we defined the list of agents inhibiting and inducing CYP3A metabolism as well as other drugs with synergistic/antagonistic effects on clarithromycin or statins to detect co-administration of each one (as confounder of the effect of clarithromycin on statins) during the observation period (Additional file 1). We used prescription of other systemic antibiotics (ATC code-J01) during the observation period as proxy for the severity of disease. We compared the frequency of the outcome between exposed persons and unexposed controls. Baseline data, demographics and risk or exposure related covariates were tabulated and compared using two-sided hypothesis tests.

### Statistical analyses

We used a generalised linear regression model to assess the association between the outcome and the exposure to statins with a log-link function to get directly risk ratios (RR) as the measure of effect. For the main analysis, the exposure was current concomitant use of CYP3A4 metabolised statins and the outcome was death or hospitalisation within 30 days. The unit of analysis for these models was a course of clarithromycin treatment. We allowed for within person correlation in case of repeated treatment courses by using robust standard errors. To adjust for potential confounding, we also included candidate covariates if they changed the main effect by >10 % in a bivariate analysis and if they were not considered moderator variables. We tested for first order interactions by including interaction terms into the models. We performed several sensitivity analyses to assess the robustness of our estimates. Accordingly, we repeated our analyses using several definitions of exposure status by exposure time and statin type. We also analysed hospitalisation and mortality separately. Likewise, the analyses have been repeated by allowing for varying the outcome time definitions. We also used several modelling approaches (including logistic regression and proportional hazards regression) and methods for handling correlated data (robust standard errors or random effects models).

Data analysis was performed using Microsoft Excel and Stata 11 (Stata Corp, College Station, TX). A two-sided *p*-value <0.05 was considered statistically significant.

## Additional file

Additional file 1:
**Drug Interactions for clarithromycin and statins.** Synergistic Interactions with Clarithromycin. Major Inducers of CYP3A4. Synergistic Interactions with Simvastatin, Lovastatin, Pravastatin, Fluvastatin, Atorvastatin, Rosuvastatin. Antagonistic Interaction with Simvastatin, Lovastatin, Pravastatin, Fluvastatin, Atorvastatin. (PDF 470 kb)

## Abbreviation

AERR: Adverse event reporting rate and ratio
BGKK: Regional Sickness Fund of Burgenland
DDD: Defined Daily Dose
ICD: International Classification of Diseases
THIN: The UK Health Improvement Network
