# Supplementary material for: A population-based analysis of the risk of drug interaction between clarithromycin and statins for hospitalisation or death
Source: Lipids Health Dis. 2015 Oct 24;14:131. doi: 10.1186/s12944-015-0134-y (PMC4619489; doi:10.1186/s12944-015-0134-y)
Supplement: Additional file 1: — Drug Interactions for clarithromycin and statins. Synergistic Interactions with Clarithromycin. Major Inducers of CYP3A4. Synergistic Interactions with Simvastatin, Lovastatin, Pravastatin, Fluvastatin, Atorvastatin, Rosuvastatin. Antagonistic Interaction with Simvastatin, Lovastatin, Pravastatin, Fluvastatin, Atorvastatin. (PDF 470 kb) [file 12944_2015_134_MOESM1_ESM.pdf]

ADDITIONAL FILE

A population-based analysis of the risk of drug interaction between clarithromycin and statins for hospitalisation or death

Bita Mesgarpour[1,2], Ghazaleh Gouya[1], Harald Herkner[3], Berthold Reichardt[4], Michael Wolzt[1*]

1 Department of Clinical Pharmacology, Medical University of Vienna, Vienna, Austria

2 Digestive Diseases Research Institute (DDRI), Tehran University of Medical Sciences (TUMS), Tehran, Iran

3 Department of Emergency Medicine, Medical University of Vienna, Vienna, Austria

4 Sickness Fund Burgenland, Eisenstadt, Austria

***Corrosponding Author:** Michael Wolzt, MD

Department of Clinical Pharmacology, Allgemeines Krankenhaus Wien, Medical University of Vienna, Währinger Gürtel 18-20, 1090 Vienna, Austria

Tel: +43 (0)1 40400 29810

Fax: +43 (0)1 40400 29980

Email: michael.wolzt@meduniwien.ac.at

Drug Interactions for clarithromycin and statins

Synergistic Interactions with Clarithromycin*

Severe		Moderate		Weak	
Antiarrhythmics	C01B	Abirateron	L02BX03	Acrivastine[5]	R06AX18
HMG-CoA reductase inhibitors[1]	C10AA	Alfentanil[4]	N01AH02	Aprepitant[3]	A04AD12
Cisapride[2,3]	A03FA02	Alfuzosin[2]	G04CA01	Benzodiazepine	N05BA N05CD
Colchicine	M04AC01	Aliskiren	C09XA02	Benzodiazepine-like hypnotics	N05CF
Dronedarone[2,3]	C01BD07	Amiodarone[2]	C01BD01	Bilastine[5]	R06AX29
Droperidol[2]	N05AD08	Amisulpride[2]	N05AL05	Bortezomib	L01XX32
Eletriptan[3]	N02CC06	Amitriptyline[2]	N06AA09	Brentuximab vedotin[8]	L01XC12
Ergot alkaloids	N02CA	Antiprotozoals	P01	Cetirizine[5]	R06AE07
Ivabradin	C01EB17	Arsenic trioxide[2]	L01XX27	Cinacalcet	H05BX01
Pimozide[3]	N05AG02	Artenimol+piperaquine[2]	P01BF05	Contraceptives	G03A
Quetiapin[2]	N05AH04	Astemizole[2,3,5]	R06AX11	Darifenacin	G04BD10
Ranolazine[2]	C01EB18	Atazanavir[2]	J05AE08	Desloratadine[5]	R06AX27
Sertindole[2]	N05AE03	Azithromycin[2]	J01FA10	Ebastine[5]	R06AX22
Ticagrelor	B01AC24	Bedaquiline[2]	J04AK05	Fexofenadine[5]	R06AX26
		Bepridil[2]	C08EA02	Fidaxomicin[9]	A07AA12
		Budesonide[3]	R03BA02	HIV-Protease Inhibitors	J05AE
		Buspirone	N05BE01	Levocetirizine[5]	R06AE09
		Carboxamide derivatives	N03AF	Loratadine[5]	R06AX13
		Chloral hydrate[2]	N05CC01	Mirtazapin[2]	N06AX11
		Chloroquine[2]	P01BA01	Mizolastine[5]	R06AX25
		Chlorpromazine[2]	N05AA01	Nateglinide[¶]	A10BX03
		Ciclosporin[3]	L04AD01	Nevirapin	J05AG01 J05AR05 J05AR07
		Cilostazol	B01AC23	Non-nucleoside reverse transcriptase inhibitors	J05AG
		Ciprofloxacin[2]	J01MA02	Oxybutynin	G04BD04
		Citalopram[2]	N06AB04	Pioglitazone[¶]	A10BG03
		Clomipramine[2]	N06AA04	Repaglinid	A10BX02
		Clozapine[2]	N05AH02	Rupatadine[5]	R06AX28
		Conivaptan[3]	C03XA02	Ruxolitinib[10]	L01XE18
		Dabigatran	B01AE07	Sulfonylurea derivatives	A10BB
		Dapoxetin	G04BX14	Tolterodin	G04BD07
		Darifenacin[3]	G04BD10	Trazodon	N06AX05
		Darunavir[3]	J05AE10	Vemurafenib	L01XE15

	Dasatinib[3]	L01XE06	Verapamil and its analogue	C08DA
	Desipramine[2]	N06AA01	Vitamin K antagonists	B01AA
	Digoxin and its derivatives	C01AA	Warfarin[¶]	B01AA03
	Diphenhydramine[2]	R06AA02		
	Disopyramide[2]	C01BA03		
	Dofetilide[2]	C01BD04		
	Dolasetron[2]	A04AA04		
	Domperidone[2]	A03FA03		
	Doxepin[2]	N06AA12		
	Eplerenone[3]	C03DA04		
	Eribulin[2]	L01XX41		
	Erythromycin[2]	J01FA01		
	Escitalopram[2]	N06AB10		
	Everolimus[3]	L04AA18		
	Famotidine[2]	A02BA03		
	Felbamate[2]	N03AX10		
	Felodipine[3]	C08CA02		
	Fentanyl, combinations[4]	N01AH51		
	Fentanyl[3,4]	N01AH01		
	Fesoterodin	G04BD11		
	Fingolimod[2]	L04AA27		
	Flecainide[2]	C01BC04		
	Fluconazole[2]	J02AC01		
	Fluoxetine[2]	N06AB03		
	Fluticasone[3]	R03BA05<br>R01AD08		
	Foscarnet[2]	J05AD01		
	Fosphenytoin[2]	N03AB05		
	Galantamine[2]	N06DA04		
	Gatifloxacin[2]	J01MA16		
	Granisetron[2]	A04AA02		
	Halofantrine[2]	P01BX01		
	Haloperidol[2]	N05AD01		
	Ibutilide[2]	C01BD05		
	Iloperidone[2]	N05AX14		
	Imipramine[2]	N06AA02		
	Immunosuppressant	L04A		
	Indapamide[2]	C03BA11		
	Indinavir[3]	J05AE02		
	Insulin[¶]	A10AB		
	Isradipine[2]	C08CA03		
	Itraconazole[2]	J02AC02		
	Ivacaftor[3,7]	R07AX02		
	Ketoconazole[2]	J02AB02		
	Lapatinib[2]	L01XE07		

	Levofloxacin[2]	J01MA12
	Levomethadyl[2]	N07BC05
	Lithium[2]	N05AN
	Lopinavir[3]	J05AR10
	Lurasidone[3]	N05AE05
	Maraviroc[3]	J05AX09
	Mesoridazine[2]	N05AC03
	Methadone[2]	N07BC02
	Methylprednisolone[¶]	H02AB04
	Midazolam[3]	N05CD08
	Moexipril/HCTZ[2]	C09BA13
	Moxifloxacin[2]	J01MA14
	Neuroleptika (antipsychotics)	N05AD
	Nicardipine[2]	C08CA04
	Nilotinib[2]	L01XE08
	Nisoldipine[3]	C08CA07
	Nortriptyline[2]	N06AA10
	Ofloxacin[2]	J01MA01
	Olanzapine[2]	N05AH03
	Omeprazole[¶]	A02BC01<br>A02BD01<br>A02BD05
	Ondansetron[2]	A04AA01
	Opioids	N02A
	Oxytocin[2]	H01BB02
	Paliperidone[2]	N05AX13
	Paroxetine[2]	N06AB05
	Pasireotide[2]	H01CB05
	Pentamidine[2]	P01CX01
	Perflutren lipid microspheres[2]	V08DA01
	Pimozide[2]	N05AG02
	Probucol[2]	C10AX02
	Procainamide[2]	C01BA02
	Promethazine[2]	R06AD02
	Protein kinase inhibitors	L01XE
	Protriptyline[2]	N06AA11
	Quetiapine[3]	N05AH04
	Quinidine[2,3]	C01BA01
	Quinine sulfate[2]	P01BC01
	Reboxetin	N06AX18
	Remifentanil[4]	N01AH06
	Rifabutin	J04AB04
	Rilpivirine[2]	J05AG05
	Risperidone[2]	N05AX08
	Ritonavir[2]	J05AE03<br>J05AR10
	Roxithromycin*[2]	J01FA06

	Salmeterol	R03AC12
	Saquinavir[2,3]	J05AE01
	Sertraline[2]	N06AB06
	Sevoflurane[2]	N01AB08
	Sildenafil[3,6]	G04BE03
	Sirolimus[3]	L04AA10
	Solifenacin[2]	G04BD08
	Sotalol[2]	C07AA07
	Sparfloxacin[2]	J01MA09
	Sufentanil[4]	N01AH03
	Sunitinib[2]	L01XE04
	Tacrolimus[2,3]	L04AD02
	Tadalafil[6]	G04BE08
	Tamoxifen[2]	L02BA01
	Taxanes	L01CD
	Telavancin[2]	J01XA03
	Telithromycin[2]	J01FA15
	Temsirolimus	L01XE09
	Theophylline and its derivatives	R03DA
	Terfenadine[2,3,5]	R06AX12
	Thioridazine[2]	N05AC02
	Tipranavir[3]	J05AE09
	Tizanidine[2]	M03BX02
	Tolterodine[2]	G04BD07
	Tolvaptan[3]	C03XA01
	Trazodone[2]	N06AX05
	Triazolam[3]	N05CD05
	Tricyclic antidepressants and their analogues	N06AA
	Trimethoprim-Sulfamethoxazole[2]	J01EE01
	Trimipramine[2]	N06AA06
	Vandetanib[2]	L01XE12
	Vardenafil[2,3,6]	G04BE09
	Venlafaxine[2]	N06AX16
	Vinblastine[¶]	L01CA01
	Vinorelbine[¶]	L01CA04
	Voriconazole[2]	J02AC03
	Vorinostat[2]	L01XX38
	Zidovudine	J05AF01<br>J05AR01<br>J05AR04<br>J05AR05
	Ziprasidone[2]	N05AE04

* The list is adopted from the table of interaction in *medis* (Medikamenten-Informations-Systems) for clarithromycin and clarithromycin package insert.

[¶] The interaction is not addressed in *medis* but in clarithromycin product information.

[1]"Cholestrol-Synthese-Hemmer", albeit excluding fluvastatin and rosuvastatin which are not dependent on CYP3A metabolism.

[2]"Stoffe, die QT-Zeit-veräng. können", adopted from the list of drugs that prolong the QT interval and/or induce Torsades de Pointes (http://www.azcert.org/)

[3] CYP3A substrates, adopted from FDA http://www.fda.gov/Drugs/DevelopmentApprovalProcess/DevelopmentResources/DrugInteractionsLabeling/ucm093664.htm#cypEnzymes

[4] fentanyl and its derivatives

[5] H1-antihistamines (second- generation)

[6] Phosphodiesterase-5 inhibitors

[7] Ivacaftor is a sensitive CYP3A substrate. Co-administration with ketoconazole, a strong CYP3A inhibitor, significantly increased ivacaftor exposure [measured as area under the curve (AUC)] by 8.5-fold (Ivacaftor product information). AUC increase by clarithromycin is predicted 4.35 fold by using method presented by Hisaka 2009.

[8] Co-administration of brentuximab vedotin, primarily metabolized by CYP3A, with ketoconazole increased exposure to brentuximab vedotin by approximately 34% (ADCETRIS™ (brentuximab vedotin) product information). AUC increase by clarithromycin is predicted 1.28 fold by using method presented by Hisaka 2009.

[9] Co-administration of single doses of the P-gp inhibitor cyclosporine A and DIFICLIR in healthy volunteers, resulted in a 4- and 2-fold increase in fidaxomicin Cmax and AUC, respectively and in a 9.5 and 4-fold increase in Cmax and AUC, respectively, of the main active metabolite OP-1118. As the clinical relevance of this increase in exposure is unclear, co-administration of potent inhibitors of P-gp, such as cyclosporine, ketoconazole, erythromycin, clarithromycin, verapamil, dronedarone and amiodarone are not recommended (DIFICLIR®(fidaxomicin tablet) product information).

[10] The Cmax and AUC of ruxolitinib increased 33% and 91%, respectively, with Jakafi administration (10 mg single dose) following ketoconazole 200 mg twice daily for four days, compared to receiving ruxolitinib alone in healthy subjects (JAKAFI™ (ruxolitinib) product information). AUC increase by clarithromycin is predicted 1.69 fold by using method presented by Hisaka 2009.

Major Inducers of CYP3A4* (antagonistic DDI)

Carbamazepine	N03AF01
Phenytoin	N03AB02
Rifampicin	J04AB02
Rifabutin	J04AB04
Phenobarbital	N03AA02

* The list is adopted from Polasek 2011. Bosentan, Efavirenz and Modafinil are excluded from the list because they are also metabolized by CYP3A.

Synergistic Interactions with Simvastatin*

Major		Moderate		Minor	
Danazol¶	G03XA01	Amiodarone	C01BD01	Amlodipine	C08CA01
Imidazole antifungal[1]	J02AB	Colchicine	M04AC01	Boceprevir	J05AE12
Macrolides	J01FA	Daptomycin	J01XX09	Cilostazol	B01AC23
Nefazodone[2]¶	N06AX06	Digoxin¶	C01AA05	Dronedarone	C01BD07
Telaprevir	J05AE11	Diltiazem¶	C08DB01	Fenofibrate	C10AB05
Triazole antifungal[1]	J02AC	Fibrates	C10AB	Imatinib	L01XE01
		Fusidic acid	J01XC01	Nicotinic acid and derivatives	C04AC
		HIV-Protease Inhibitors	J05AE	Stiripentol	N03AX17
		Immunosuppressant	L04A	Vitamin K antagonists	B01AA
		Ranolazin	C01EB18		
		Ticagrelor	B01AC24		
		Verapamil and its analogue	C08DA		

* The list is adopted from the table of interaction in *medis* (Medikamenten-Informations-Systems) for simvastatin and simvastatin package insert.

¶ The interaction is not addressed in *medis* but in simvastatin product information.

[1] Azole-antifungals

[2] When single 40-mg doses of simvastatin, substrates of CYP3A4, was given to healthy adult volunteers who had received nefazodone 200 mg BID for 6 days, approximately 20-fold increases in plasma concentrations of simvastatin and simvastatin acid were seen (SERZONE® (nefazodone) product information)

Antagonistic Interaction with Simvastatin

Bosentan[1]	C02KX01
Cholestyramine[2]	C10AC01
Colestipol[3]	C10AC02
Etravirine[4]	J05AG04
Rifampin[5]	J04AB02

[1] Bosentan is an endothelin receptor antagonist. Coadministration of bosentan decreased the plasma concentrations of simvastatin (a CYP3A substrate), and its active β-hydroxy acid metabolite, by approximately 50%. The plasma concentrations of bosentan were not affected (TRACLEER® (bosentan) product information).

[2] Cholestyramine is a strongly basic "ion-exchange resin". An in vivo animal experiment demonstrated a significant reduction (about 50% with AUC) in the concentration of active hydroxy acid form of simvastatin in plasma following the co-administration of simvastatin (500 mg/kg p.o.) and cholestyramine (600 mg/kg p.o.), compared with the administration of simvastatin alone (Nakai 1996).

[3] Colestipol is a is a weakly basic "ion-exchange resin" and may combine with simvastatin in the stomach and prevent it from being absorbed properly into the body.

[4] Lovastatin and simvastatin are CYP3A substrates and co-administration with INTELENCE® may result in lower plasma concentrations of the HMG-CoA reductase inhibitor (INTELENCE®(etravirine) product information).

[5] Rifampin decreased the total area under the plasma concentration-time curve of simvastatin and simvastatin acid by 87% ($P < .001$) and 93% ($P < .001$), respectively. Also the peak concentrations of both simvastatin and simvastatin acid were reduced greatly (by 90%) by rifampin ($P < .001$) (Kyrklund 2000).

Synergistic Interactions with Lovastatin*

Major		Moderate		Minor	
Danazol¶	G03XA01	Amiodarone	C01BD01	Boceprevir	J05AE12
Imidazole antifungal[1]	J02AB	Colchicine	M04AC01	Cilostazol	B01AC23
Macrolides	J01FA	Daptomycin	J01XX09	Dronedarone	C01BD07
Nefazodone¶	N06AX06	Diltiazem¶	C08DB01	Fenofibrate	C10AB05
Telaprevir	J05AE11	Fibrates	C10AB	Imatinib	L01XE01
Triazole antifungal[1]	J02AC	Fusidic acid	J01XC01	Nicotinic acid and derivatives	C04AC
		HIV-Protease Inhibitors	J05AE	Stiripentol	N03AX17
		Immunosuppressant	L04A	Vitamin K antagonists	B01AA
		Ranolazin¶	C01EB18		
		Ticagrelor	B01AC24		
		Verapamil and its analogue	C08DA		

* The list is adopted from the table of interaction in *medis* (Medikamenten-Informations-Systems) for lovastatin and lovastatin package insert.

¶ The interaction is not addressed in *medis* but in lovastatin product information.

[1] Azole-antifungals

Antagonistic Interaction with Lovastatin

Bosentan[1]	C02KX01
Etravirine[2]	J05AG04
Rifampin	J04AB02

[1] Bosentan is an endothelin receptor antagonist. It is expected to reduce plasma concentrations of other statins that are significantly metabolized by CYP3A, such as lovastatin and atorvastatin (TRACLEER® (bosentan) product information).

[2] Lovastatin and simvastatin are CYP3A substrates and co-administration with INTELENCE® may result in lower plasma concentrations of the HMG-CoA reductase inhibitor (INTELENCE®(etravirine) product information).

Synergistic Interactions with Pravastatin*

Major	ATC code	Moderate	ATC code	Minor	ATC code
Ciclosporin	L04AD01	Boceprevir	J05AE12	Cimetidine[1]	A02BA01
Macrolides	J01FA	Colchicine	M04AC01	Eltrombopag	B02BX05
		Daptomycin	J01XX09	Fenofibrate	C10AB05
		Fibrates	C10AB	Nicotinic acid and derivatives	C04AC
		HIV-Protease Inhibitors	J05AE	Verapamil[2]	C08DA01
		Immunosuppressant	L04A		
		Rifampicin	J04AB02		
		Teriflunomid	L04AA31		

* The list is adopted from the table of interaction in *medis* (Medikamenten-Informations-Systems) for pravastatin and pravastatin package insert.

[1] Cimetidine (300 mg QID for 3 days) plus pravastatin (20 mg single dose) increased area under the serum concentration-time curve (AUC) of pravastatin by 30% (PRAVACHOL®(rosuvastatin calcium) product information).

[2] Verapamil (Verapamil IR 120 mg for 1 day and Verapamil ER 480 mg for 3 days) plus pravastatin (40 mg single dose) increased area under the serum concentration-time curve (AUC) of pravastatin by 31% (PRAVACHOL®(rosuvastatin calcium) product information).

Antagonistic Interaction with Pravastatin

Cholestyramine[1]	C10AC01
Colestipol[2]	C10AC02

[1] Cholestyramine (24 g OD for 4 weeks) plus pravastatin (20 mg BID for 8 weeks) decreased area under the serum concentration-time curve (AUC) of pravastatin by 51% (PRAVACHOL®(rosuvastatin calcium) product information).

[2] Colestipol (10 g single dose) plus pravastatin (20 mg single dose) decreased area under the serum concentration-time curve (AUC) of pravastatin by 47% (PRAVACHOL®(rosuvastatin calcium) product information).

Synergistic Interactions with Fluvastatin*

Major	Moderate		Minor	
	Colchicine	M04AC01	Diclofenac[¶2]	M01AB05
	Daptomycin	J01XX09	Etravirine[3]	J05AG04
	Fibrates	C10AB	Fenofibrate	C10AB05
	Imidazole antifungal[1]	J02AB	Glibenclamide[¶4]	A10BB01
	Immunosuppressant	L04A	Nicotinic acid and derivatives[¶5]	C04AC
	Triazole antifungal[1]	J02AC	Phenytoin[¶6]	N03AB02
			Stiripentol	N03AX17
			Vitamin K antagonists	B01AA

* The list is adopted from the table of interaction in *medis* (Medikamenten-Informations-Systems) for fluvastatin and fluvastatin package insert.

¶ The interaction is not addressed in *medis* but in fluvastatin product information.

1 Azole-antifungals

2 Diclofenac (25 mg QD) plus fluvastatin (40 mg QD for 8 days) increased systemic exposure of fluvastatin and diclofenac by 50% and 25%, respectively (Lescol® (fluvastatin sodium) product information).

3 Fluvastatin is metabolized by CYP2C9 and co-administration with INTELENCE® may result in higher plasma concentrations of the HMG-CoA reductase inhibitor. Dose adjustments for these HMG-CoA reductase inhibitors may be necessary (INTELENCE®(etravirine) product information).

4 Glibenclamide (5- 20 mg QD for 22 days) plus fluvastatin (40 mg b.i.d. for 21 days) increased systemic exposure of fluvastatin and glibenclamide by 51% and 70%, respectively (Lescol® (fluvastatin sodium) product information).

5 The risk of skeletal muscle effects may be enhanced when LESCOL is used in combination with lipid-modifying doses (≥1 g/day) of niacin; a reduction in LESCOL dosage should be considered in this setting (Lescol® (fluvastatin sodium) product information).

6 Phenytoin (300 mg QD) plus fluvastatin (40 mg b.i.d. for 5 days) increased systemic exposure of fluvastatin and phenytoin by 40% and 29%, respectively (Lescol® (fluvastatin sodium) product information).

Antagonistic Interaction with Fluvastatin

Bosentan[1]	C02KX01
Cholestyramine	C10AC01
Colestipol[2]	C10AC02
Rifampin[3]	J04AB02

1 Fluvastatin is a CYP2C9 substrate and it demonstrates inhibitory effects on this isoenzyme in vitro and in vivo (Scripture 2001). The Summary of Product Characteristics (SmPC) for Bosentan states ‘concomitant administration of both a CYP3A4 inhibitor and a CYP2C9 inhibitor should be avoided’.

2 Cholestyramine (8g) plus fluvastatin 20 mg QD administered 4 hrs after a meal decreased systemic exposure of fluvastatinby 51% (Lescol® (fluvastatin sodium) product information).

3 Rifampicin 600 mg QD for 6 days plus Fluvastatin (20 mg QD) decreased systemic exposure of fluvastatin by 53% (Lescol® (fluvastatin sodium) product information).

Synergistic Interactions with Atorvastatin*

Major		Moderate		Minor	
Macrolides	J01FA	Amiodarone	C01BD01	Boceprevir	J05AE12
Telaprevir	J05AE11	Colchicine	M04AC01	Oral contraceptives	G03A
		Daptomycin	J01XX09	Digitalis glycosides	C01AA
		Fibrates	C10AB	Dronedarone	C01BD07
		Fusidic acid	J01XC01	Fenofibrate	C10AB05
		HIV-Protease Inhibitors	J05AE	Imatinib	L01XE01
		Imidazole antifungal[1]	J02AB	Nicotinic acid and derivatives	C04AC
		Immunosuppressant	L04A	Stiripentol	N03AX17
		Nefazodone[2¶]	N06AX06	Vitamin K antagonists	B01AA
		Triazole antifungal[1]	J02AC		
		Verapamil and its analogue	C08DA		

* The list is adopted from the table of interaction in *medis* (Medikamenten-Informations-Systems) for atorvastatin and atorvastatin package insert.

[¶] The interaction is not addressed in *medis* but in nefazodone product information.

[1] Azole-antifungals

[2] When single 40-mg doses of atorvastatin, substrate of CYP3A4, was given to healthy adult volunteers who had received nefazodone 200 mg BID for 6 days, approximately 3- to 4-fold increases in plasma concentrations of atorvastatin and atorvastatin lactone were seen (SERZONE® (nefazodone) product information)

Antagonistic Interaction with Atorvastatin

Bosentan[1]	C02KX01
Etravirine[2]	J05AG04
Rifampin[3]	J04AB02

[1] Bosentan is an endothelin receptor antagonist. It is expected to reduce plasma concentrations of other statins that are significantly metabolized by CYP3A, such as lovastatin and atorvastatin (TRACLEER® (bosentan) product information).

[2] The combination of etravirine and atorvastatin can be given without dose adjustments, however, the dose of atorvastatin may need to be altered based on clinical response (INTELENCE®(etravirine) product information)

[3] In a controlled randomized study, ten patients received 600 mg rifampin for 5 days and a single 40-mg dose of atorvastatin on day 6. Rifampin reduced the total area under the plasma concentration-time curve (AUC) of unchanged atorvastatin (acid) by 80% (95% CI, 73% to 84%; P < .001), that of the active metabolites 2-hydroxyatorvastatin acid by 43% (95% CI, 29% to 51%; P < .001) and 4-hydroxyatorvastatin acid by 81% (95% CI, 74% to 84%; P < .001) (Backman 2005).

Synergistic Interactions with Rosuvastatin*[1]

Major		Moderate		Minor	
Ciclosporin	L04AD01	Antacids	A02A	Dronedarone[¶2]	C01BD07
		Colchicine	M04AC01	Eltrombopag	B02BX05
		Daptomycin	J01XX09	Ezetimibe[¶3]	C10AX09
		Fibrates	C10AB	Fenofibrate	C10AB05
		HIV-Protease Inhibitors	J05AE	Nicotinic acid and derivatives	C04AC
				Vitamin K antagonists	B01AA

* The list is adopted from the table of interaction in *medis* (Medikamenten-Informations-Systems) for rosuvastatin and rosuvastatin package insert.

[¶] The interaction is not addressed in *medis* but in rosuvastatin product information.

[1] Results from in vitro and in vivo studies show that rosuvastatin is neither an inhibitor nor an inducer of cytochrome P450 isoenzymes. In addition, rosuvastatin is a poor substrate for these isoenzymes. No clinically relevant interactions have been observed between rosuvastatin and either fluconazole (an inhibitor of CYP2C9 and CYP3A4) or ketoconazole (an inhibitor of CYP2A6 and CYP3A4). Concomitant administration of itraconazole (an inhibitor of CYP3A4) and rosuvastatin resulted in a 28% increase in AUC of rosuvastatin. This small increase is not considered clinically significant. Therefore, drug interactions resulting from cytochrome P450-mediated metabolism are not expected (Summary of Product Characteristics (SmPC) presented by EMEA for rosuvastatin).

[2] Dronedarone (400 mg BID) plus rosuvastatin (10 mg) increased area under the serum concentration-time curve (AUC) of rosuvastatin by 1.4 fold (CRESTOR®(rosuvastatin calcium) product information).

[3] Ezetimibe (10 mg QD, 14 days) plus rosuvastatin (10 mg QD, 14 days) increased area under the serum concentration-time curve (AUC) of rosuvastatin by 1.2 fold (CRESTOR®(rosuvastatin calcium) product information).

References

Backman 2005

Backman JT, Luurila H, Neuvonen M, Neuvonen PJ. Rifampin markedly decreases and gemfibrozil increases the plasma concentrations of atorvastatin and its metabolites. *Clin Pharmacol Ther* 2005;78(2):154-67.

Hisaka 2009

Hisaka A, Kusama M, Ohno Y, Sugiyama Y, Suzuki H. A proposal for a pharmacokinetic interaction significance classification system (PISCS) based on predicted drug exposure changes and its potential application to alert classifications in product labelling. *Clin Pharmacokinet* 2009;48(10):653-66.

Kyrklund 2000

Kyrklund C, Backman JT, Kivistö KT, Neuvonen M, Laitila J, Neuvonen PJ. Rifampin greatly reduces plasma simvastatin and simvastatin acid concentrations. *Clin Pharmacol Ther* 2000;68(6):592-7.

Nakai 1996

Nakai A, Nishikata M, Matsuyama K, Ichikawa M. Drug interaction between simvastatin and cholestyramine in vitro and in vivo. *Biol Pharm Bull* 1996;19(9):1231-3.

Polasek 2011

Polasek TM, Lin FP, Miners JO, Doogue MP. Perpetrators of pharmacokinetic drug-drug interactions arising from altered cytochrome P450 activity: a criteria-based assessment. *Br J Clin Pharmacol* 2011;71(5):727-36.
